# Do teachers have more health problems? Results from a French cross-sectional survey

**DOI:** 10.1186/1471-2458-6-101

**Published:** 2006-04-21

**Authors:** Viviane Kovess-Masféty, Christine Sevilla-Dedieu, Carmen Rios-Seidel, Eléna Nerrière, Christine Chan Chee

**Affiliations:** 1 MGEN Foundation for Public Health, 3 square Max Hymans, 75748 Paris Cedex 15, France

## Abstract

**Background:**

Although only a few studies have been published on teachers' health, certain ideas are widely accepted, such as for example, the preconceived notion that teachers suffer from an excessively high rate of mental health problems. The objective of this study is to compare teachers' mental and physical health to that of a control group.

**Methods:**

A cross-sectional postal survey was conducted among a sample of 3,679 teachers and 1,817 non-teachers aged 20 to 60 years old.

**Results:**

No lifetime prevalence of any psychiatric disorder (with the exception of undifferentiated somatoform disorder in men) or mean scores of psychological distress were found to be significantly higher in teachers. However, multiple analyses, adjusted for all confounding variables, revealed a higher risk of lifetime anxiety disorders in male teachers. On the other hand, significant differences were observed for some physical ailments: a higher lifetime prevalence of rhinopharyngitis/laryngitis in both male and female teachers, of conjunctivitis and lower urinary tract infection in male teachers and of bronchitis, eczema/dermatitis and varicose veins in female teachers. No significant difference was found for chronic pain between the two groups.

**Conclusion:**

Teachers do not seem to have poorer mental health. However, their physical condition is characterized by a higher prevalence of health problems related to the ENT tract, and to a lesser extent, depending on the gender, to skin, eyes, legs and lower urinary tract.

## Background

The idea that teachers suffer from an excessively high rate of mental health problems is widely accepted among not only the general public, but among teachers themselves [1]. Teachers report they are exposed to a high risk of stress and occupational "burnout" [2] (i.e., syndrome of emotional exhaustion and cynicism that occurs frequently among individuals who do "people-work" [3]) which they claim leads them to suffer from psychiatric disorders more than the average. However, this seems to run contrary to well-established epidemiological data in psychiatry which show that the middle classes (where the majority of teachers fall) are relatively better protected against psychiatric disorders than underprivileged classes of society where the highest prevalence rates are found [4-7]. In contrast, when it comes to their physical health, we could assume that teachers have potentially healthy life styles, although it could be expected that due to their working conditions, they may have a higher risk for certain ailments such as voice disorders or venous diseases.

### Mental health

Although a person's socioprofessional category is acknowledged to be a decisive factor in his or her mental health [5,6,8], very few studies have dealt with the differences in the prevalence of mental health problems according to occupation [9-11]. Among the different occupations analyzed, those studies that have covered teachers have reported contradictory results. The majority of studies describe a very high level of mental fatigue among teachers (i.e., for example psychological distress and burnout), connected to the specific aspects of their profession, however none of these studies have shown an excessively high rate of psychiatric disorders as defined in mental health diagnostic scales (i.e., for example, mood and anxiety disorders or alcohol abuse), in this specific occupational group.

Studies on teachers' mental health can be divided into two groups. The first group includes studies involving a wide variety of professions, among which teachers, and the second group includes studies on teachers only, with or without a control group.

The Eaton et al. study [9] is one of the few studies that falls into the first group. This study was done using data from the ECA (Epidemiologic Catchment Area Program) survey conducted in the United States in 1980 and it covered the prevalence of depression (prevalence DSM-III over the previous year using the Diagnostic Interview Schedule – DIS [12,13]) with a sample of 11,789 persons aged 18 to 64 working full time. A very detailed list of codes for occupations corresponding to 502 categories in the 1980 U.S. census was used. In all five locations where the survey was taken, the overall prevalence of depression ranged from 3% to 5%. According to their analyses, adjusted for sociodemographic variables such as age, gender, level of education and race or ethnic background, the majority of the groups of teachers surveyed (n = 520) did not suffer from a rate of depression (DIS) that was higher than the prevalence reported in the entire population (ECA).

The second group includes a larger number of studies, among which only two studies using a control group [14,15].

The Finlay-Jones study [14] used the General Health Questionnaire (GHQ) [16,17] on a random sample of teachers working for the Western Australia Department of Education. The results reported a 17% rate of acute psychological distress, which was much higher than the 9% level found in the general population in the Canberra study [18]. Only one-third of the subjects reported that they were "not exposed to high levels of stress-related factors" and among this group, the level of acute psychological distress was 11% (i.e., closer to the 9% level found in the random sample of the middle-class Australian population).

In the De Heus and Diekstra study [15], a sample of teachers (n= 1,018) was compared to a group of workers in "social-oriented" occupations (n = 2,740) (i.e., who had all stated they worked with people), drawn from a much larger pool of 13,555 volunteers. Several measuring systems were used, including a psychological distress scale (HSC) [19], the Maslach's burnout scale [3], and other items involving psychological symptoms, work-related stress and social support. This study showed that teachers were particularly vulnerable to occupational burnout, and especially male teachers whose scores on all the scales were higher than those of men engaging in other occupations. For female teachers, however, the results were not as clear-cut.

A number of other studies on teachers can be found in the literature which lack control groups and are limited to factors such as stress and psychological distress. According to these studies, teachers apparently showed quite a high level of stress and burnout [20-24]. Four main factors were identified as sources of stress: student misbehavior, poor working conditions, time pressure and poor school ethos [20]. A low level of supervision and colleague support also appeared to be related to work stress and burnout [21].

### Physical health

As in the case of mental health, only a few studies of varying quality have been published on teachers' physical health. When considering the main classes of diagnoses of physical diseases (musculoskeletal, respiratory, cardiovascular, nervous and hormonal disorders), Seibt et al. [25] showed no significant difference between the health status of teachers (secondary school) and office workers. Moreover, when focusing on cardiovascular disorders, a study carried out in Germany [26] even showed that there was a lower risk for male teachers compared to men working in 12 other professions.

However, some studies have demonstrated that for small number of more specific physical disorders, the prevalence is clearly higher for teachers. Impaired phonation represents the most characteristic teachers' physical disorder because it is directly related to their specific occupational demands when teaching. According to a number of studies aimed at identifying which occupational groups were at an increased risk of suffering from occupational voice disorders [27,28], teachers were found to be particularly vulnerable to developing such problems. Smith et al. [29] showed that compared to a control group, teachers were significantly more likely to report having 6 voice symptoms, among which hoarseness was the most frequent, and 5 related physical discomfort symptoms (tiring, effortful, ache, uncomfortable and rough).

Probably because of their occupational environment, characterized by permanent contact with people and particularly with children, the Lerman et al. study [30] opened the door to the idea that teachers could be at a higher risk of developing infectious diseases by documenting a significant increased risk of Hepatitis A infection in Israel among kindergarten staff and teachers in comparison to the general population.

It is worth mentioning that there are a few additional studies that have shown a different impact of a few other diseases on teachers: an excessive rate of some major cancers, in particular breast [31,32] and thyroid [33] cancers and surprisingly enough, an association between school teaching and mortality from autoimmune diseases [34].

The study presented here was aimed at determining if the teaching profession differs from other occupations in its impact on workers' mental and physical health by comparing the prevalence of a wide variety of mental health disorders and level of psychological distress, together with the prevalence of several physical ailments in teachers to a control group. This comparison was made possible by way of a cross-sectional postal survey conducted among persons covered by French health care insurance provided by the MGEN (*Mutuelle Générale de l'Education Nationale*). This health care insurance covers everyone working in France within the national education system or in certain ministries and research institutes, as well as his or her relatives (3 million people). The MGEN thus manages health insurance for all teachers in the public system, who make up 65% of the MGEN's clientele, the remainder often being civil servants in schools or diverse ministries, who formed a quasi ideal control group since they are very similar to teachers on the sociological level: mainly middle-class civil servants, who just like teachers, have job and income security.

## Methods

### Sample

Between June 1999 and March 2000, we carried out an epidemiological survey on the population of MGEN policyholders. A self-rated questionnaire was sent by mail to 10,000 persons aged 20 to 60 inclusive, selected at random using the national health plan records of policyholders living in continental France. Three reminder notices were sent to those who had not responded. The response rate obtained was respectively 39.4% on the first run, 23% on the second run and 26% on the third run for an overall response rate of 66.5% (n = 6,518).

The sample was weighted for all issues related to sample design in order to ensure a truly representative sampling of the population of MGEN policyholders. All analyses conducted were performed using the weighted population.

Since the objective of this study was to determine whether there was a connection between being a teacher and having health problems, we only used respondents who were actually employed and excluded job seekers, retirees and unemployed persons. Housewives were not included in the analyses, nor were non-working students. In contrast, the respondents included in this study could be healthy workers, as well as workers on sick leave. It should be noted that at the time health insurance coverage is provided by the MGEN, no information on current mental and physical health is gathered. The analyses were thus conducted on a weighted total of 5,496 employed respondents, of which 3,679 teachers and 1,817 non-teachers. Persons qualified as teachers had to actually be giving classes to students.

### Instruments

The self-administered, anonymous questionnaire covered questions on general health and use of the health care system, as well as sick days and sick leave.

The sociodemographic variables included gender, age, family composition, educational level, place of residence and naturally included a precise description of employment and occupational status. A number of questions delved into quality of life, social network and activities, as well as feelings about work.

The parameters used to measure physical and mental health are summarized in Table 1.

To measure mental health, we opted for a dual approach. First, we used questions based on the CIDIS (Composite International Diagnostic Interview Simplified [13,35]) as a diagnostic tool for mental health problems, using the self-administered version (CIDI-SA), which allows for Axis I (i.e., clinical disorders and other conditions that may be a focus of clinical attention) diagnoses according to the DSM-IV criteria standardized diagnostic instrument (DSM-IV) [36]. Second, we used a scale widely validated in the international literature to measure psychological distress: the Hopkins Symptom Checklist (HSC), Ilfeld's version (the Psychiatric Symptom Index (PSI)) [37-39]. This scale is composed of 29 questions that measure symptoms of anxiety, distress and depression occurring over the last week. Psychotic disorders (i.e., delusions and prominent hallucinations) were not included in our survey considering how rarely they occur within the working population and due to the fact our questionnaire was self-administered. In addition we used CAGE screening to evaluate the risk of alcohol abuse [40].

Physical health was studied through two series of questions: a list of illnesses presented by subtypes (cardiovascular, endocrine, etc.) and a list of chronic symptoms (i.e., lasting 6 months or longer), such as diverse types of pain that could be involved in somatoform disorders (i.e., disorders characterized by physical complaints that appear to be medical in origin but that cannot be explained in terms of a physical disease).

To measure disease frequency, we used period prevalence, which represents the proportion of the sample that are cases at any time within a stated period. Two reference periods were used to determine prevalence rates: lifetime and the twelve previous months. The prevalence rates of both mental and physical health problems are presented for a lifetime period. One-year prevalence was used in multiple analysis only.

### Data analysis

We used the chi-square test to compare the sociodemographic and employment features of the teacher and control groups. Given the distortion observed between some of the basic features of the two groups, Mantel-Haenszel's chi-square tests were used to compare ratios and prevalence rates, and linear regression analyses were applied to compare mean values.

Multiple logistic regression models were used to measure the effect of a panel of sociodemographic and employment variables on the presence of a psychiatric disorder.

Statistical processing was performed using SPSS 13.0 software.

## Results

### Sociodemographic and employment features

Table 2 shows the sociodemographic and employment (i.e., career cycle and working environment) features of the teacher and control groups. The two groups differed on most of the variables except marital status and long-term sick leave. Teachers were more often female, higher educated and relatively younger, although they had more seniority in their current profession. Teachers more often had an irregular work schedule and lived closer to their work place.

### Mental health

#### Lifetime prevalence of psychiatric disorders

Table 3 shows the lifetime prevalence of the diverse DSM-IV psychiatric disorders by gender among both teacher and control groups. The significant differences were very few. Male teachers had a higher prevalence of undifferentiated somatoform disorders (3.1% vs. 1.7%), whereas anorexia nervosa was found to be more prevalent among control group women (0.6% vs. 0.2%).

#### Factors associated with the presence of a mental health disorder

Multiple analyses were used to measure the effect of occupation on the presence of a mental health disorder on a lifelong and one-year basis for both men (Table 4) and women (Table 5). We adjusted our models for the sociodemographic and employment variables which significantly differed between both groups (see Table 2). Whereas no occupational impact was found for most disorders either over lifetime or in the last year, a higher risk of lifetime anxiety disorders was found in male teachers.

With regard to sociodemographic features, ageing in men was clearly associated with an increased risk for lifetime anxiety and alcohol disorders. Living alone appeared to be a factor leading to a higher risk for both men and women of all disorders, except for alcohol disorders in men. Having completed graduate level training was associated with a lower risk of depressive disorders in both men and women and of anxiety disorders in women only. Low educational level showed a different pattern depending on the gender and diagnosis. Not having completed a baccalaureate degree was associated with a decreased risk for depressive disorders in men, whereas a higher risk for anxiety disorders was observed in women.

As regards employment features, work schedule had no significance. The number of years in current profession showed a different pattern depending on the gender and diagnosis. Men with less seniority showed a higher risk of anxiety disorders, whereas the opposite results were found for women. Moreover, higher seniority in women appeared to be clearly associated with an increased risk for lifetime depressive disorders. A commute from home to work of over one hour was clearly associated with a higher risk of lifetime anxiety disorders for both genders. It was also associated with a higher risk of lifetime depression in women only.

#### Levels of psychological distress

There was no significant difference in mean scores between the two groups (Teachers: 11.2; Controls: 11.3; p > 0.05).

As expected, there was a significant difference in mean scores between the two genders (Men: 8.6; Women: 13.3; p < 0.001), but no significant interaction between occupation and gender was found (p > 0.05).

An increase in psychological distress with age was observed. For the group in the 20–39 age bracket, the mean score was 9.6 (SD = 9.9). For subjects aged 40–49, the score was 11.8 (SD = 10.4) and for those 50 and older, the score reached 12.3 (SD = 11.4). Here, a significant interaction between age and occupation was found (p < 0.01). Ageing in teachers was clearly associated with a higher psychological distress score (p < 0.001), while no difference was identified in controls (p > 0.05).

#### Life satisfaction levels and social networks

As regards life satisfaction, teachers reported they were more satisfied with their lives than non-teachers when it came to aspects such as housing (p < 0.001), environment (p < 0.05), amount (p < 0.001) and use (p < 0.01) of free time. However, in the area of human relations, no significant difference in terms of level of satisfaction in their relationships with their children, parents, friends or in their love life was observed.

As regards social activities, such as meeting family members, going out with friends, participating in clubs and volunteer organizations or practicing sports, no significant difference in frequency (p > 0.05) was found between teacher and control groups.

### Physical health

#### Body mass index

Table 6 presents mean BMI (BMI Quetelet formula) values by gender and age. No difference was found between the two groups either in men or women (p > 0.05). However, in men, the distribution of BMI revealed less excess weight in the teacher group than in the control group: 37.6% vs. 45.6% (p < 0.05).

#### Lifetime prevalence of physical health problems

Table 7 presents the 12 most frequently reported diseases among a list of 36, as well as the 3 most frequent chronic types of pain among a list of 23.

With regard to physical health problems, lifetime prevalence of rhinopharyngitis/laryngitis was found to be higher in teachers for both genders (p < 0.001). In male teachers only, conjunctivitis and cystitis were also more prevalent (p < 0.05). For female teachers, the prevalence of bronchitis, eczema/dermatitis and varicose veins was found to be significantly higher (p < 0.05).

As for chronic pain (i.e., symptoms experienced during a period of at least six months over lifetime), the three most reported types of pain were: back pain (roughly one-third of respondents), articular pain and headaches. No significant difference between the two groups was found in either gender.

## Discussion

To date, only a few studies of varying quality have been published on teachers' health. The present study is one of the very few studies comparing teachers' health – both physical and mental – to that of an appropriate control group.

The results of this study would tend to indicate that teachers do not seem to have poorer mental health. No lifetime prevalence of any psychiatric disorder (with the exception of undifferentiated somatoform disorder in men) or mean scores of psychological distress were found to be significantly higher in teachers. However, multiple analyses, adjusted for all confounding variables, revealed a higher risk of lifetime anxiety disorders in male teachers. On the other hand, in terms of physical health, significant differences were found between teacher and control groups, with higher lifetime prevalence in teachers of rhinopharyngitis/laryngitis in both genders, of conjunctivitis and lower urinary tract infection in men and of bronchitis, eczema/dermatitis and varicose veins in women. No significant difference between teacher and control groups was found for chronic pain.

This study shows that the preconceived notion that teachers have a higher risk of developing mental health problems is apparently a myth. First, as reported in previous publications [9], this study shows that teachers do not generally have a higher rate of psychiatric disorders. Second, one major finding of this study is that contrary to other results previously published [20-24], teachers do not seem to suffer more from psychological distress. However, their level of psychological distress does grow with age. Compared to non-teachers, they seem to be more satisfied with their living conditions (housing, environment or free time).

In contrast, this study does show that there is a higher prevalence in teachers of a number of physical disorders. The most affected organs are in the ENT tract. The throat, in particular, is affected and several studies have already reported that teachers seem to be prone to such problems, particularly those related to voice [27-29]. Other physical disorders appear to be more prevalent in teachers as well. To our knowledge, the present study is the first to report these results. These findings are, however, not so easy to interpret since they concern only one gender. According to the literature, some of these health problems may be related to certain working conditions experienced by teachers: exposure to chalk dyes as a possible cause for contact dermatitis [41-43], standing at work as a predisposing factor for varicose veins [44-46], and work constraints [47,48] as a reason for adopting at-risk habits for urinary tract infections, such as suppressing the desire to urinate or drinking less to avoid needing to use the toilet (as has already been observed in teachers [49]).

There are, however, several limitations to this study. First, health problems are self-reported. Reporting on minor or past events is likely to be particularly prone to anamnestic error since information was collected for a lifetime period. This could result in an underestimated prevalence of disorders. On the other hand, as regards physical health, an approximate knowledge of medical terms, together with long lists of physical disorders could potentially result in some erroneous answers. However, this normally would not have any impact on the results of our comparison. Second, selecting the controls from the population of MGEN policyholders could introduce a source of potential bias, as some of them, essentially the headmasters or school inspectors, could be former teachers. However, Table 8 shows that this represents only a small minority of the control subjects. Third, although there is no selection bias due to sick leave, as the teacher group included teachers who were working, as well as those who had stopped working for health reasons, a possible selection bias could, however, result from the fact that this group did not include teachers who had changed their profession. Fourth, 33.5% of the persons selected for this survey decided not to complete the questionnaire. Their health status may be different from that of the respondents and this could also introduce a potential bias. Fifth, we did not take into account all the characteristics of the working environment that could introduce a source of potential bias in our comparison (especially, working time and tenure). Sixth, in this survey, no information was collected on the number of times one individual had been affected by a given physical disorder. This could be of interest, especially to compare frequent illnesses, such as urinary tract infections in women, for example. Finally, we did not determine which occupational risk factors specific to the teaching profession could be associated with the higher frequency of some diseases observed in teachers.

Nevertheless, a good number of the results of the present study are important for the potential they have in terms of public health and policy implications, especially because some easy to implement measures to change conditions or behavior at work could definitely be of help to improve on prevention of some of the health problems identified. Better information for teachers and adjusting their training (for example, advice given by a speech therapist to encourage good voice habits) and workplace conditions (for example, short breaks and adequate toilet facilities) could certainly improve teachers' behavior, which would very likely have an impact on the occurrence of the disease.

## Conclusion

This study shows that teachers do not seem to have poorer mental health, although multiple analyses revealed a higher risk of lifetime anxiety disorders in male teachers. In contrast, their physical condition is characterized by a higher prevalence of health problems related to the ENT tract, and to a lesser extent, depending on the gender, to skin, eye, leg and lower urinary tract disorders. In our study, no analysis was done to identify potential at-risk subgroups in teachers or the specific occupational factors that could be related to the increased prevalence observed in teachers for certain diseases. Further studies, preferably prospective, are thus required to thoroughly pursue research in this area. This could be a step forward to improve on prevention of some of the occupational-related diseases in teachers, especially if easy to implement measures could be recommended to modify potential at-risk conditions or habits at work.

## Abbreviations

BMI Body Mass Index

CAGE Cut down, Annoyed, Guilty, Eye-opener

CIDIS Composite International Diagnostic Interview Simplified

CIDI-SA Composite International Diagnostic Interview – Self-Administered

DIS Diagnostic Interview Schedule

DSM Diagnostic and Statistical Manual of mental disorders (III: third edition, IV: fourth edition)

ECA Epidemiologic Catchment Area

ENT Ear, Nose and Throat

GHQ General Health Questionnaire

HSC Hopkins Symptom Checklist

MDE Major Depressive Episode

PSI Psychiatric Symptom Index

## Competing interests

The author(s) declare that they have no competing interests.

## Authors' contributions

Viviane Kovess-Masféty, MD, PhD, designed the study and participated in drafting the manuscript. Christine Sevilla-Dedieu, PhD, contributed substantively by extensively revising the initial draft of the manuscript (statistical analysis, interpretation of the data and writing). Carmen Rios-Seidel, MD, performed statistical analysis, interpreted the data and managed the initial drafting of the manuscript. Eléna Nerrière, PhD, assisted in extensively revising the initial draft of the manuscript. Christine Chan Chee, MD, participated in designing and implementing the study.

## Pre-publication history

The pre-publication history for this paper can be accessed here:



## Figures and Tables

**Table 1:** List of parameters used to measure mental and physical health

**Health problems**	**Measuring instruments**
**Mental health**	
Psychological distress	Ilfeld's version of the Hopkins Symptom Checklist (HSC): the Psychiatric Symptom Index (PSI)
Mood disorders, Anxiety disorders, Alcohol abuse/dependency disorders, Anorexia nervosa, Bulimia, Somatization disorders, Undifferentiated somatoform disorders	CIDI-SA according to DSM-IV criteria
Alcohol abuse	CAGE screening test
**Physical health**	
Ophthalmologic diseases; Cardiovascular diseases; Osteo-articular diseases; Oto Rhino Laryngological diseases; Diseases of the digestive system; Diseases of the breathing apparatus; Diseases of the nervous system; Endocrinological diseases and diseases of the metabolism; Diseases of the mouth and the teeth; Diseases of the genital-urinary bodies; Skin diseases; Insomnia; Other diseases	a series of questions concerning physical illnesses over lifetime (in case of a positive answer, a question was asked about any current episode)
Migraines; Nausea; Difficulties in swallowing; Vomiting; Diarrhoea; Bloating; Bad taste in the mouth; Abdominal pains (apart from menstruation); Back pains; Articular pains; Feeling of losing the breath (apart from physical effort); Chest pains; Palpitations or spasmophilia; Extremity pains; Marks or discoloration of the skin; Numbness or itching; Muscular weakness; Frequent urge to urinate or difficulties in urinating; Burning feeling around the mouth or the sexual organs; Periods of amnesia of a few hours or days; Sensation of loss of balance; Fainting; Fuzzy, double or scrambled vision a few times (apart from a problem with glasses); Excessively painful menstrual periods; Unusual or abundant vaginal discharge	a series of questions concerning chronic symptoms over lifetime (lasting 6 months or longer)

**Table 2:** Sociodemographic and employment features of the teacher and control groups

**Features**	**Teachers (N = 3,679)**		**Controls (N = 1,817)**		**p**
			
	**n**	**%**	**n**	**%**	
**Sociodemographic**					
Gender					0.000***
Male	1,496	40.7	875	48.2	
Female	2,183	59.3	942	51.8	
					
Age					0.003**
20 – 39 years♣	1,191	32.5	541	30.1	
40 – 49 years◆	1,216	33.2	553	30.8	
≥ 50 years♠	1,259	34.3	702	39.1	
					
Marital status					0.056
Married or living with someone	2,866	78.2	1,392	76.9	
Single	467	12.7	213	11.8	
Separated or divorced	285	7.8	176	9.7	
Widowed	45	1.2	28	1.5	
					
Educational level					0.000***
Lower than Baccalaureate degree	48	1.3	674	37.5	
Baccalaureate degree	675	18.3	273	15.2	
≤ 2 years at university	724	19.7	273	15.2	
3 – 4 years at university	1,582	43.0	339	18.9	
≥ 5 years at university	650	17.7	239	13.3	
					
**Career cycle**					
Number of years in current profession					0.000***
< 5 years	417	11.4	315	17.9	
5 – 9 years	473	12.9	259	14.7	
10 – 19 years	723	19.8	370	21.0	
20 – 29 years	1,173	32.1	502	28.5	
≥ 30 years	873	23.9	315	17.9	
					
Long-term sick leave					0.509
At least one in the past five years	96	2.6	42	2.3	
None	3,583	97.4	1,774	97.7	
					
**Working environment**					
Work schedule					0.000***
Irregular	1,860	51.2	668	37.8	
Regular	1,770	48.8	1,099	62.2	
					
Commuting time from home to work					0.000***
≤ 15 minutes	1,228	34.0	586	34.2	
16 – 30 minutes	1,194	33.1	500	29.2	
31 – 60 minutes	831	23.0	382	22.3	
> 60 minutes	356	9.9	243	14.2	

n = number of cases in each category for each group. % = proportion of cases in each category for each group. p = p-value from Chi-square test (*p < 0.05; **p < 0.01; ***p < 0.001). Mean age of the subjects in each group: ♣ Teacher: 32.1, Control: 31.4 ; ◆ Teacher: 44.8, Control: 45.3 ; ♠ Teacher: 53.3, Control: 53.6.

**Table 3:** Comparison of lifetime prevalence of mental disorders (DSM-IV) by gender between teacher and control groups

**Lifetime DSM-IV diagnoses**	**Males**						**Females**					
		
	**Teachers (N = 1,496)**			**Controls (N = 875)**			**Teachers (N = 2,183)**			**Controls (N = 942)**		
		
	**N**	**n**	**%**	**N**	**n**	**%**	**N**	**n**	**%**	**N**	**n**	**%**
**Mood disorders**												
Major Depressive Episode (MDE)	1,455	337	23.2	835	173	20.7	2,116	862	40.8	863	348	40.3
MDE moderate	1,455	163	11.2	835	84	10.1	2,116	424	20.0	863	179	20.7
MDE severe	1,455	58	4.0	835	34	4.1	2,116	214	10.1	863	95	11.0
Other mood disorders^†^	1,468	179	12.2	847	98	11.6	2,142	453	21.1	885	166	18.8
Any mood disorder	1,455	373	25.6	835	195	23.4	2,116	943	44.6	863	372	43.1
												
**Anxiety disorders**												
Social phobia	1,469	29	2.0	850	27	3.2	2,109	64	3.0	876	43	4.9
Specific phobia	1,468	37	2.5	835	27	3.2	2,114	87	4.1	868	53	6.1
Agoraphobia	1,470	66	4.5	850	29	3.4	2,110	237	11.2	874	115	13.2
Agoraphobia without history of panic disorder	1,470	56	3.8	850	27	3.2	2,110	222	10.5	874	103	11.8
Any phobia (social, specific or agoraphobia)	1,457	94	6.5	834	47	5.6	2,081	306	14.7	860	140	16.3
Panic disorder without agoraphobia	1,441	9	0.6	829	4	0.5	2,095	19	0.9	866	7	0.8
Panic disorder with agoraphobia	1,441	5	0.3	829	1	0.1	2,095	13	0.6	866	11	1.3
Any panic disorder (with or without agoraphobia)	1,441	15	1.0	829	5	0.6	2,095	34	1.6	866	18	2.1
Obsessive-Compulsive Disorder (OCD)	1,434	15	1.0	824	6	0.7	2,080	18	0.9	860	8	0.9
Any anxiety disorder (phobia, panic disorder or OCD)	1,417	109	7.7	807	52	6.4	2,017	330	16.4	820	152	18.5
												
**Other disorders**												
Alcohol abuse	1,445	13	0.9	844	8	0.9	2,045	8	0.4	842	3	0.4
Alcohol dependency	1,445	16	1.1	844	9	1.1	2,045	8	0.4	842	6	0.7
CAGE ≥ 2	1,429	176	12.3	818	92	11.2	1,948	86	4.4	756	48	6.3
Anorexia nervosa	1,485	0	0.0	791	0	0.0	2,152	5	0.2	850	5	0.6*
Bulimia	1,413	17	1.2	815	13	1.6	2,018	100	5.0	872	47	5.4
Somatization disorder	1,439	2	0.1	801	4	0.5	2,081	23	1.1	862	13	1.5
Undifferentiated somatoform disorder	1,439	44	3.1	801	14	1.7*	2,081	76	3.7	862	45	5.2

N = Total weighted number of respondents in each category for each group. n = number of cases in each category for each group. % = proportion of cases in each category for each group. p = p-value from Mantel-Haenszel chi-square test adjusted for age and educational level (*p < 0.05 ; **p < 0.01 ; ***p < 0.001). ^† ^Other mood disorders: adjustment disorder with depressed mood acute (<6 months), chronic (> 6 months) and dysthymic disorder.

**Table 4:** Factors influencing the probability of having a mental disorder among males (N = 2,371)

**Factors**	**Major Depressive Episode (MDE)**				**Anxiety disorder**				**CAGE ≥ 2**			
			
	**Lifetime**^†^		**One year**^‡^		**Lifetime**^†^		**One year**^‡^		**Lifetime**^†^		**One year**^‡^	
			
	**OR**	**95% CI**	**OR**	**95% CI**	**OR**	**95% CI**	**OR**	**95% CI**	**OR**	**95% CI**	**OR**	**95% CI**
**Sociodemographic features**												
												
Age												
*20 – 39 years*	*1.00*		*1.00*		*1.00*		*1.00*		*1.00*		*1.00*	
40 – 49 years	1.97	1.40–2.77***	1.73	0.99–2.98	3.54	2.03–6.18***	2.28	1.06–4.91*	2.68	1.72–4.17***	1.88	0.54–6.63
≥ 50 years	1.34	0.89–2.02	0.60	0.29–1.22	3.99	2.07–7.70***	1.68	0.62–4.56	2.72	1.62–4.56***	0.78	0.16–3.76
												
Marital status												
*Married or living with someone*	*1.00*		*1.00*		*1.00*		*1.00*		*1.00*		*1.00*	
Single	1.81	1.30–2.50***	1.35	0.78–2.34	2.03	1.21–3.42**	2.64	1.35–5.18**	1.24	0.80–1.93	2.57	0.90–7.35
Separated or divorced	1.65	1.08–2.52*	1.15	0.55–2.41	1.59	0.84–3.02	1.19	0.40–3.50	0.62	0.31–1.23	0.52	0.07–4.14
Widowed	1.42	0.28–7.07	5.99	1.08–33.3*	4.18	0.75–23.35	0.00	-	0.36	0.02–7.60	0.00	-
												
Educational level												
Lower than Baccalaureate degree	0.58	0.38–0.87**	0.92	0.46–1.84	1.56	0.81–2.99	1.04	0.36–2.98	1.41	0.85–2.32	0.49	0.12–2.07
Baccalaureate degree	0.82	0.57–1.20	1.08	0.56–2.09	1.38	0.76–2.52	1.80	0.78–4.17	0.91	0.55–1.49	0.51	0.12–2.12
≤ *2 years at university*	*1.00*		*1.00*		*1.00*		*1.00*		*1.00*		*1.00*	
3 – 4 years at university	0.91	0.68–1.23	1.48	0.88–2.50	1.08	0.64–1.84	1.24	0.59–2.59	0.80	0.53–1.20	0.92	0.33–2.55
≥ 5 years at university	0.68	0.49–0.96*	1.35	0.75–2.42	0.77	0.42–1.42	0.52	0.21–1.34	0.85	0.55–1.32	1.14	0.38–3.46
												
**Career cycle features**												
Number of years in current profession												
*< 5 years*	*1.00*		*1.00*		*1.00*		*1.00*		*1.00*		*1.00*	
5 – 9 years	1.01	0.68–1.52	0.91	0.44–1.87	1.00	0.50–1.98	2.65	1.01–6.95*	1.55	0.91–2.64	1.74	0.31–9.82
10 – 19 years	0.99	0.66–1.47	1.38	0.71–2.69	0.81	0.42–1.54	1.54	0.55–4.29	0.71	0.41–1.24	2.41	0.44–13.34
20 – 29 years	0.72	0.46–1.12	1.30	0.62–2.75	0.41	0.20–0.84*	0.88	0.28–2.76	0.68	0.38–1.22	3.61	0.57–22.77
≥ 30 years	0.87	0.52–1.47	1.50	0.60–3.77	0.35	0.15–0.79*	1.07	0.28–4.03	0.55	0.28–1.07	3.30	0.36–30.63
												
**Working environment features**												
Work schedule												
Irregular	0.94	0.75–1.17	0.82	0.57–1.19	0.69	0.47–1.01	1.09	0.64–1.87	1.10	0.82–1.48	0.76	0.34–1.69
*Regular*	*1.00*		*1.00*		*1.00*		*1.00*		*1.00*		*1.00*	
Commuting time from home to work												
≤ *15 minutes*	*1.00*		*1.00*		*1.00*		*1.00*		*1.00*		*1.00*	
16 – 30 minutes	1.10	0.84–1.44	1.07	0.68–1.70	1.36	0.86–2.14	1.38	0.74–2.57	1.15	0.81–1.62	0.37	0.10–1.40
31 – 60 minutes	1.45	1.10–1.90**	1.58	1.02–2.45*	1.35	0.84–2.17	1.07	0.54–2.12	1.23	0.86–1.76	2.49	1.06–5.85*
> 60 minutes	1.31	0.93–1.86	0.87	0.45–1.65	1.86	1.07–3.22*	1.05	0.44–2.48	1.17	0.75–1.84	0.79	0.19–3.22
**Occupational group**												
Teacher	1.03	0.80–1.34	0.82	0.54–1.26	1.74	1.10–2.73*	1.55	0.79–3.03	1.41	0.99–1.98	0.66	0.28–1.55
*Control*	*1.00*		*1.00*		*1.00*		*1.00*		*1.00*		*1.00*	

All variables included in the logistic regression analyses are mentioned in the table. Only subjects with no data missing for any of the variables were included in these models. ^† ^Existence of the disease during lifetime. ^‡ ^Existence of the disease during the previous year. OR = odd ratio. 95% CI = 95% confidence interval. *p < 0.05; **p < 0.01; ***p < 0.001.

**Table 5:** Factors influencing the probability of having a mental disorder among females (N = 3,125)

**Factors**	**Major Depressive Episode (MDE)**				**Anxiety disorder**				**Cage ≥ 2**			
			
	**Lifetime**^†^		**One year**^‡^		**Lifetime**^†^		**One year**^‡^		**Lifetime**^†^		**One year**^‡^	
			
	**OR**	**95% CI**	**OR**	**95% CI**	**OR**	**95% CI**	**OR**	**95% CI**	**OR**	**95% CI**	**OR**	**95% CI**
**Sociodemographic features**												
												
Age												
*20 – 39 years*	*1.00*		*1.00*		*1.00*		*1.00*		*1.00*		*1.00*	
40 – 49 years	0.89	0.69–1.17	0.97	0.67–1.40	1.18	0.83–1.68	0.71	0.43–1.17	1.50	0.80–2.81	3.91	0.57–26.63
≥ 50 years	0.94	0.69–1.29	0.91	0.58–1.43	1.23	0.82–1.86	0.49	0.26–0.92*	1.68	0.81–3.45	0.65	0.07–6.16
												
Marital status												
*Married or living with someone*	*1.00*		*1.00*		*1.00*		*1.00*		*1.00*		*1.00*	
Single	1.36	1.07–1.72*	1.52	1.11–2.08**	1.36	1.01–1.85*	1.24	0.82–1.88	1.27	0.68–2.35	7.37	1.97–27.52**
Separated or divorced	2.30	1.80–2.95***	1.38	0.98–1.93	1.46	1.06–2.00*	1.82	1.17–2.83**	2.89	1.83–4.57***	16.09	5.52–46.87***
Widowed	2.00	1.16–3.44*	0.85	0.35–2.07	1.62	0.84–3.11	3.09	1.38–6.94**	0.00	-	0.00	-
												
Educational level												
Lower than Baccalaureate degree	0.95	0.68–1.33	1.15	0.70–1.88	1.74	1.14–2.64*	0.76	0.40–1.47	1.07	0.52–2.23	1.07	0.17–6.66
Baccalaureate degree	0.98	0.76–1.25	1.08	0.74–1.58	1.02	0.74–1.42	0.97	0.61–1.54	0.56	0.31–1.02	0.09	0.01–0.69*
≤ *2 years at university*	*1.00*		*1.00*		*1.00*		*1.00*		*1.00*		*1.00*	
3 – 4 years at university	0.98	0.78–1.23	1.26	0.90–1.75	0.86	0.63–1.16	0.70	0.46–1.06	0.67	0.40–1.13	0.04	0.01–0.22***
≥ 5 years at university	0.65	0.48–0.87**	1.26	0.83–1.92	0.62	0.41–0.94*	0.76	0.45–1.29	0.74	0.40–1.42	0.61	0.20–1.87
												
**Career cycle features**												
												
Number of years in current profession												
*< 5 years*	*1.00*		*1.00*		*1.00*		*1.00*		*1.00*		*1.00*	
5 – 9 years	1.32	0.97–1.80	1.33	0.86–2.04	0.57	0.37–0.89*	0.34	0.19–0.63***	1.58	0.63–3.96	0.06	0.00–0.86*
10 – 19 years	1.37	1.01–1.85*	1.37	0.89–2.09	0.98	0.66–1.46	0.64	0.39–1.06	1.97	0.84–4.65	0.02	0.00–0.40*
20 – 29 years	1.71	1.21–2.42**	1.45	0.89–2.37	1.00	0.64–1.57	0.76	0.41–1.40	2.01	0.81–5.01	0.38	0.07–2.16
≥ 30 years	1.74	1.16–2.59**	1.17	0.66–2.09	1.05	0.63–1.76	0.94	0.44–2.01	1.52	0.54–4.27	0.38	0.04–3.53
												
**Working environment features**												
												
Work schedule												
Irregular	1.02	0.86–1.21	0.94	0.74–1.20	1.09	0.87–1.36	1.31	0.96–1.79	0.92	0.61–1.38	1.62	0.60–4.40
*Regular*	*1.00*		*1.00*		*1.00*		*1.00*		*1.00*		*1.00*	
												
Commuting time from home to work												
≤ *15 minutes*	*1.00*		*1.00*		*1.00*		*1.00*		*1.00*		*1.00*	
16 – 30 minutes	0.83	0.69–1.01	0.83	0.63–1.10	0.97	0.76–1.25	1.29	0.90–1.85	1.75	1.10–2.78*	4.50	1.47–13.82**
31 – 60 minutes	1.05	0.85–1.30	1.15	0.85–1.55	1.06	0.80–1.41	1.04	0.68–1.58	1.41	0.82–2.42	0.09	0.00–2.85
> 60 minutes	1.40	1.06–1.85*	1.42	0.98–2.07	1.48	1.04–2.11*	1.16	0.68–1.96	1.46	0.72–2.96	1.20	0.15–9.80
												
**Occupational group**												
												
Teacher	1.01	0.82–1.25	1.03	0.76–1.40	1.12	0.85–1.49	1.01	0.68–1.49	0.99	0.59–1.65	2.06	0.50–8.47
*Control*	*1.00*		*1.00*		*1.00*		*1.00*		*1.00*		*1.00*	

All variables included in the logistic regression analyses are mentioned in the table. Only subjects with no data missing for any of the variables were included in these models. ^† ^Existence of the disease during lifetime. ^‡ ^Existence of the disease during the previous year. OR = odd ratio. 95% CI = 95% confidence interval. *p < 0.05; **p < 0.01; ***p < 0.001.

**Table 6:** Mean BMI values of the teacher and control groups by gender

**Age**	**Mean values**			
	
	**Males**		**Females**	
		
	**Teachers (N = 1,496)**	**Controls (N = 875)**	**Teachers (N = 2,183)**	**Controls (N = 942)**
20 – 39 years	23.7	23.9	21.7	22.6
40 – 49 years	24.8	25.1	22.5	22.9
≥ 50 years	25.0	25.8	23.0	23.3
**Total**	**24.5**	**25.0**	**22.4**	**22.9**

BMI = Body Mass Index. Mean value = observed mean age of the subjects in each category for each group. For both males and females, a linear regression analysis was applied to test the difference in mean values between the two groups, while controlling for age and educational level. No significant difference was found.

**Table 7:** Most frequent physical health problems and chronic pain over lifetime by gender for the teacher and control groups

**Physical health**	**Males**						**Females**					
		
	**Teachers (N = 1,496)**			**Controls (N = 875)**			**Teachers (N = 2,183)**			**Controls (N = 942)**		
		
	**N**	**n**	**%**	**N**	**n**	**%**	**N**	**n**	**%**	**N**	**n**	**%**
**Physical health problems**												
Rhinopharyngitis/laryngitis	1,454	497	34.2^(1)^	838	198	23.6^(1) ^***	2,169	1,018	46.9^(1)^	919	256	27.9^(1)^***
Bronchitis	1,454	307	21.1^(2)^	838	163	19.5^(2)^	2,169	482	22.2^(5)^	919	164	17.8^(6)^*
Otitis	1,454	264	18.2^(3)^	838	155	18.5^(3)^	2,169	424	19.6^(7)^	919	147	16.0^(9)^
Eczema/dermatitis	1,454	252	17.3^(4)^	838	118	14.1^(4)^	2,169	492	22.7^(4)^	919	153	16.7^(8)^*
Arthrosis	1,454	167	11.5^(5)^	838	104	12.4^(5)^	2,169	356	16.4^(9)^	919	186	20.3^(4)^
Conjunctivitis	1,454	155	10.7^(6)^	838	61	7.3^(10)^*	2,169	419	19.3^(8)^	919	158	17.2^(7)^
Asthma	1,454	143	9.8^(7)^	838	75	8.9^(8)^	2,169	180	8.3^(12)^	919	56	6.1^(12)^
Urinary tract infection	1,454	130	8.9^(8)^	838	54	6.4^(11)^	2,169	574	26.5^(2)^	919	206	22.4^(3)^
Infections	1,454	128	8.8^(9)^	838	63	7.5^(9)^	2,169	316	14.6^(10)^	919	133	14.5^(10)^
Varicose veins	1,454	127	8.7^(10)^	838	80	9.5^(7)^	2,169	434	20.0^(6)^	919	166	18.1^(5)^*
Hypertension	1,454	119	8.2^(11)^	838	82	9.8^(6)^	2,169	220	10.1^(11)^	919	96	10.5^(11)^
Cystitis	1,454	47	3.2^(12)^	838	11	1.3^(12)^*	2,169	563	26.0^(3)^	919	220	24.0^(2)^
												
**Chronic pains**												
Back pain	1,440	444	30.8^(1)^	801	278	34.7^(1)^	2,081	782	37.6^(1)^	862	373	43.3^(1)^
Articular pain	1,440	292	20.3^(2)^	801	179	22.3^(3)^	2,081	467	22.4^(3)^	862	225	26.1^(3)^
Headache/migraine	1,440	250	17.4^(3)^	801	180	22.5^(2)^	2,081	692	33.3^(2)^	862	346	40.1^(2)^

N = Total weighted number of respondents in each category for each group. n = number of cases in each category for each group. % = proportion of cases in each category for each group. p = p-value from Mantel-Haenszel chi-square test adjusted for age and educational level (*p < 0.05 ; **p < 0.01 ; ***p < 0.001). Numbers in brackets represent the order of appearance in the ranking of physical health problems or chronic pain according to prevalence

**Table 8:** Professions of the teacher and control groups

**Professions**	**n**	**%**
**Teachers (N = 3,679)**		
Nursery school	401	10.9
Elementary school	934	25.4
Intermediate school	868	23.6
Regular secondary school	422	11.5
Vocational secondary school	464	12.6
Special education school	118	3.2
Post-secondary school	351	9.5
Other	122	3.3
		
**Controls (N = 1,817)**		
Headmaster, principal, school inspector	165	9.1
School counselor, school psychologist, documentalist	91	5.0
Professional occupation^†^	49	2.7
Craftsman, shopkeeper, company head, farmer	50	2.8
Executive, manager	334	18.4
Health professional, social worker	112	6.2
Technician, supervisor	154	8.5
Office worker, shop assistant	367	20.2
Worker, maintenance personnel	458	25.2
Other	36	1.9

n = number of cases in each category for each group. % = proportion of cases in each category for each group. ^† ^For example, physician, dentist, pharmacist, lawyer...
